# Dissecting the Niche for Alveolar Type II Cells With Alveolar Organoids

**DOI:** 10.3389/fcell.2020.00419

**Published:** 2020-06-04

**Authors:** Danying Liao, Huaibiao Li

**Affiliations:** ^1^Department of Haematology, Union Hospital, Tongji Medical College, Huazhong University of Science and Technology, Wuhan, China; ^2^Institute of Reproductive Health, Tongji Medical College, Huazhong University of Science and Technology, Wuhan, China

**Keywords:** lung, alveolar epithelium, alveolar type II epithelial cell, alveolar stem cell, alveolar organoid, Wnt pathway, FGF pathway, coronavirus

## Introduction

The self-renewal and differentiation of tissue stem cells are dictated by the microenvironment in which they reside, the so-called stem cell niche (Scadden, 2006; de Cuevas and Matunis, 2011; Chacón-Martínez et al., 2018; Pinho and Frenette, 2019). To date, the importance of the niche in maintaining tissue homeostasis is increasingly appreciated, given the capability of stem cells to restore normal tissue function upon injury (Wabik and Jones, 2015). With the advent of new techniques, such as *in vivo* imaging, lineage tracing models and single-cell sequencing, our understanding of the interaction between stem cells and the niche under both normal physiological and pathological conditions is broadened (Zepp et al., 2017; Joost et al., 2018; Nguyen and Currie, 2018). However, the complexity and dynamics of the niche within the tissue, which are difficult to recapitulate in 2D culture, compound the effort to pinpoint the contribution of each niche component to stem cell function. Here, we discuss how to deconvolute the complexity of the stem cell niche with organotypic culture methods using alveolar stem cells within the lung as an example.

**Figure 1:**
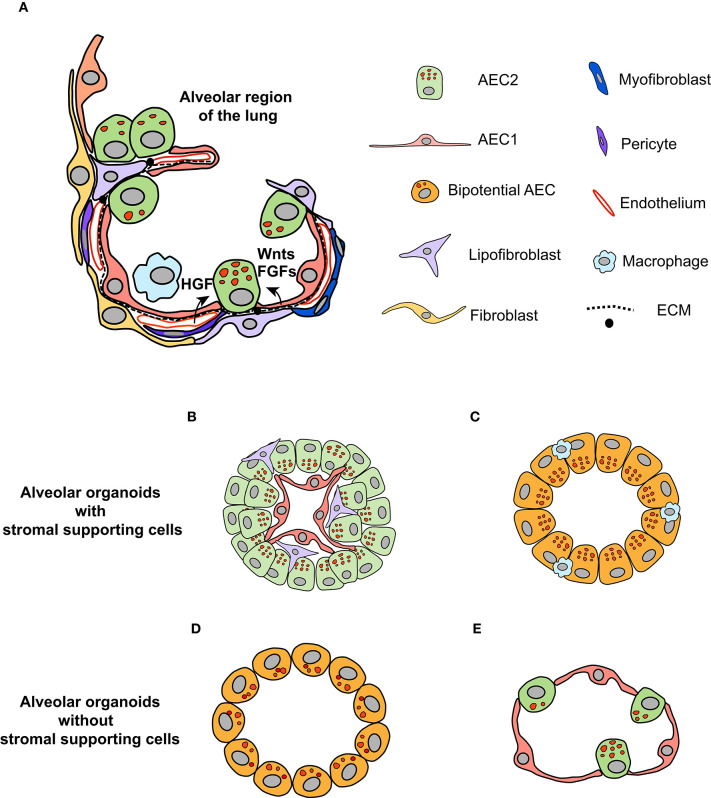
Organotypic culture to dissect the role of the niche in regulating the fate of AEC2 cells. **(A)** The microenvironment in which AEC2 cells inhabit composes of different types of stromal cells within the alveoli regions of the lung, including fibroblasts, endothelial cells, pericytes, and immune cells (adapted from Barkauskas et al., 2017 with modifications). Together with AEC1 cells and ECM components, these stromal cells form the niche for AEC2 cells. The paracrine signals generated by stromal cells regulate the behavior of AEC2 stem cells during homeostasis and regeneration states. **(B,C)** Organotypic coculture of AEC2 cells with stromal cells give rise to alveolar organoids. The alveolar organoids supported by mesenchymal cells or endothelial cells have similar structure, in which AEC1 cells are surrounded by AEC2 cells, with stromal cells mingled with alveolar epithelial cells **(B)**. The alveolar organoids promoted by macrophages is mainly composed of cells positive for both the AEC2 marker (SPC) and the AEC1 marker (RAGE), suggesting that these organoids might originate from the bipotential cells **(C)**. **(D)** The alveolar organoids induced by defined culture medium, independent of stromal supporting cells. The cells within this type of organoids exhibit overlapped signals of SPC and AQP5 (the marker for AEC1 cell). **(E)** Based on the knowledge of pathways that promote AEC2 proliferation and differentiation, we propose that alveolar organoids that are similar in structure to alveoli of the lung can be stimulated by defined growth factors in a stepwise manner.

The alveoli in the distal regions of the lung are the primary sites for gas exchange (Brody and Williams, 1992). The alveolar epithelia mainly consist of type II (AEC2) and type I (AEC1) epithelial cells (Figure 1A; Brody and Williams, 1992). The latter are squamous cells responsible for gas exchange, covering most of the surface area of alveoli (Brody and Williams, 1992). AEC2 cells have cuboidal shape and maintain the stability of alveoli through synthesis and secretion of surfactant proteins (reviewed in detail by Fehrenbach, 2001; Beer and Moodley, 2017). In addition to these characteristics, AEC2 cells are proposed to be the stem cells within the alveolar epithelia (Fehrenbach, 2001; Barkauskas et al., 2013). This is supported by results from lineage tracing studies (Barkauskas et al., 2013; Desai et al., 2014; Zacharias et al., 2018). Within the normal lung, AEC2 cells are able to differentiate into AEC1 cells, albeit at a very low turnover rate (Barkauskas et al., 2013; Desai et al., 2014). Injuries to the lung trigger the rapid proliferation of AEC2 cells, followed by differentiation to AEC1 cells to restore the normal function of the lung (Barkauskas et al., 2013; Desai et al., 2014; Nabhan et al., 2018; Zacharias et al., 2018). Of note, single-cell sequencing and lineage tracing studies have unraveled the heterogeneity of AEC2 cells that display differential capacity of proliferation and differentiation in both homeostatic and regenerative states (Desai et al., 2014; Treutlein et al., 2014; Nabhan et al., 2018; Zacharias et al., 2018; Riemondy et al., 2019). Therefore, a subpopulation of AEC2 cells might serve as the stem/progenitor cells to maintain the homeostasis of the alveoli in the lung (Hogan et al., 2014).

## The Niche of AEC2 Cells

Within the alveolar epithelia, AEC2 cells are in contact with AEC1 cells via cell junctions (Fehrenbach, 2001). There are several types of stromal cells in the interstitial region, including mesenchymal cells, pericytes, endothelial cells, and immune cells (Hogan et al., 2014; Tan and Krasnow, 2016; Endale et al., 2017). Together with extracellular matrix (ECM), these cells constitute the “putative” niche for AEC2 cells (Figure 1A; Hogan et al., 2014). How the niche modulates the behavior of AEC2 cells starts to unfold, driven by lineage tracing models. Nabhan and colleagues showed that a subpopulation of AEC2 cells are Axin2-positive; these cells localize at close proximity to Wnt-expressing fibroblasts (Nabhan et al., 2018). The “juxtacrine” Wnt signal maintains the stemness of Axin2+AEC2 cells, whereas the loss-of-contact with the niche promotes their differentiation to AEC1 cells (Nabhan et al., 2018). Using single-cell RNA sequencing and reporter mouse lines, five subpopulations of mesenchymal cells are identified, based on the levels of PDGFRα, Wnt2, and Axin2 (Zepp et al., 2017). Spatial distance mapping further revealed that PDGFRα-Axin2 double-positive mesenchymal cells localize closer to AEC2 cells than other subpopulations (Zepp et al., 2017). Collectively, these results support the notion that mesenchymal cells in close contact with AEC2 cells are critical components of the alveolar stem cell niche.

Deconvolution of the niche complexity requires a reductionist system through which the contribution of a single niche component to AEC2 behavior can be examined. Organotypic culture appears to suit this purpose, in which the interaction between the stem cell and the niche can be interrogated (Kretzschmar and Clevers, 2016; Murrow et al., 2017). AEC2 cells have been cocultured with various types of stromal cells in Matrigel to form spheroids, including lung fibroblasts, endothelial cells and macrophages (Figures 1B,C, Supplementary Table 1; McQualter et al., 2010; Chen et al., 2012; Barkauskas et al., 2013; Lee et al., 2014; Lechner et al., 2017). With the support of mesenchymal cells, AEC2 cells grow into spheroids with multiple layers of cells, in which AEC1 cells are lined along the inner lumen surface, surrounded by AEC2 cells, referred to as alveolar organoids (Figure 1B; Chen et al., 2012; Barkauskas et al., 2013, 2017). It seems that the presence of mesenchymal cells promotes both the self-renewal and differentiation of AEC2 cells (Barkauskas et al., 2013). One of the tempting explanations is the proximity of AEC2 cells to mesenchymal cells, as suggested by the *in vivo* data. Indeed, when cultured on top of mesenchymal cells, the differentiation of AEC2 cells is blocked (Sucre et al., 2018). Furthermore, the capacity of organoid induction by subpopulations of mesenchymal cells is evaluated via the organotypic coculture system, among which PDGFRα-Axin2 double-positive populations show the highest efficiency (Zepp et al., 2017). Overall, the application of alveolar organoid facilitates the examination of the role of stromal cells in regulating the fate of AEC2 cells.

## Signals From the Niche Directing the Fate of AEC2 Cells

Niche-derived paracrine signals modulate the behavior of AEC2 cells, among which FGF signaling is of particular importance (Figure 1A). It has been demonstrated that FGF ligands secreted by lung fibroblasts are pivotal to AEC2 proliferation and differentiation, e.g., FGF7 and FGF10 (Fehrenbach, 2001). Deletion of FGFR2 receptor, which is highly expressed in AEC2 cells, results in loss of AEC2 cells, thereby leading to lung fibrosis (Dorry et al., 2019). In agreement with previous findings, the supplementation of FGF7 in the medium of organotypic coculture dramatically enhances the formation and size of alveolar organoids (Zepp et al., 2017). Nevertheless, FGF7 alone is insufficient to induce alveolar organoid formation in mesenchymal cell-free organotypic culture (Shiraishi et al., 2019a), implying that additional factors from mesenchymal cells are necessary to activate the proliferation of AEC2 cells. Analysis of putative ligand-receptor interactions between mesenchymal and AEC2 cells has identified the TGF-β, BMP, Wnt, and Notch pathways as those that regulate alveologenesis (Zepp et al., 2017; Shiraishi et al., 2019a). Results from organotypic coculture systems demonstrate that these pathways have distinct roles in alveologenesis. Activation of the Wnt pathway enhances the self-renewal of AEC2 cells and blocks their differentiation to AEC1 cells (Nabhan et al., 2018), while addition of BMP4 to the medium inhibits AEC2 proliferation and promotes their differentiation (Zepp et al., 2017; Chung et al., 2018).

Prior work has shown that vascular endothelium is essential for alveolization during lung development and regeneration (McGrath-Morrow et al., 2005; Ding et al., 2011; Lazarus et al., 2011), indicating that endothelial cells and pericytes are important niche components of AEC2 cells (Hogan et al., 2014; Mammoto and Mammoto, 2019), apart from mesenchymal cells. In organotypic coculture, endothelial cells also stimulate the formation of alveolar organoids (Figure 1B), through the secretion of thrombospondin-1 (Lee et al., 2014). Although not tested yet, pericytes likely have a similar effect in organotypic coculture of AEC2 cells as other cellular components, since pericytes are also sources of HGF, Wnt11, TGF-β, and BMP4 ligands (Kato et al., 2018). Similar to FGF ligands, HGF is a potent mitogen for AEC2 cells, when added in organotypic coculture (McQualter et al., 2010).

The impact of immune cells on the proliferation and differentiation of AEC2 cells has attracted increasing attention since they are recruited to the lung and release a variety of cytokines to initiate inflammatory response upon lung injury (Fehrenbach, 2001; Cohen et al., 2018). Targeted cytokine screenings with the organotypic coculture of AEC2 cells have identified cytokines that have distinct influences on alveolar organoid formation (Katsura et al., 2019; Glisinski et al., 2020). Specifically, IL-13 treatment disrupts the differentiation of AEC2 cells and reprograms the alveolar cells toward bronchiolar-like cells (Glisinski et al., 2020). In contrast, other cytokines, including IL-1, IL-6, and TNF-α, enhance the proliferation of AEC2 cells without inhibiting their differentiation, thereby increasing the growth of alveolar organoids (Zepp et al., 2017; Katsura et al., 2019). The presence of mesenchymal cells in coculture compounds the effort to determine whether the effects of these cytokines on AEC2 behavior are direct or indirect. Therefore, the role of cytokines in modulating AEC2 behavior can be further verified through mesenchymal cell-free alveolar organoids.

Recently, progress has been made to use defined growth factors and inhibitors to stimulate the growth of alveolar organoids (Supplementary Table 1; Shiraishi et al., 2019a,b; Weiner et al., 2019). The supplementation of Notch ligands (Jagged1 and Noggin), KGF, GSK-β inhibitor (CHIR-99021), and ALK5 inhibitor (SB431542) in the culture medium replaces the mesenchymal cells to stimulate alveolar organoid formation (Figure 1D; Shiraishi et al., 2019a,b). Of note, the cells within these organoids display overlapped signals of the AEC2 marker SPC and the AEC1 marker AQP5 (Shiraishi et al., 2019a). One explanation is that the cocktail of growth factors and inhibitors reprograms the AEC2 cells to a bipotential state (Treutlein et al., 2014). Furthermore, the AEC2-like cells isolated from these organoids are unable to differentiate to AEC1 cells when transplanted into bleomycin-injured lung (Weiner et al., 2019), suggesting that simultaneous modulation of multiple pathways likely impairs the differentiation capacity of AEC2 cells. Thus, the composition of culture medium requires further optimization for supporting cell-free organotypic culture in the future. On the other hand, mesenchymal-free organotypic culture of AEC2 cells implies that it is feasible to stimulate the growth of alveolar organoids that are similar in structure to alveoli within the lung (Figure 1E). We propose that the expansion of AEC2 cells can be initially activated by mitogens, such as FGFs and HGF, followed by activation of the BMP pathway to promote the differentiation of AEC2 cells (Chung et al., 2018).

## Modeling the Interaction Between AEC2 Cells and the Niche With Alveolar Organoids

Alveolar organoids can be employed to elucidate the reciprocal interaction between AEC2 cells and the niche in a pathological context (Fiorini et al., 2020; Li et al., 2020). For instance, the dysfunction of AEC2 cells is regarded as the driver of pulmonary fibrosis, in which aberrant deposition of collagen produced by the mesenchymal cells is one of the main manifestations (Martinez et al., 2017; Parimon et al., 2020). Apart from ECM, the cellular composition of the niche also changes in the fibrotic lung, in which seven subtypes of mesenchymal cell are identified by single-cell sequencing, with increased percentage of matrix fibroblasts, compared to the normal lung (Booth et al., 2012; Xie et al., 2018). How do these changes impact the function of AEC2 cells? Alveolar organoids allow us to examine the contribution of a niche component to AEC2 dysfunction by adding the fibrosis-associated niche components into the housing matrix. To date, Matrigel, the main component of which is laminin, collagen IV, and entactin (Li et al., 2016), is widely used as the housing matrix for organotypic culture of AEC2 cells (Supplementary Table 1). The laminin-rich Matrigel can promote the growth of integrin-high AEC2 cells to form alveolar organoids (Chapman et al., 2011). Thus, to minimize variation due to the heterogeneity of AEC2 cells, it is recommended to also examine the change in AEC2 behavior through reseeding alveolar organoids in a housing matrix containing fibrosis-associated niche components. Additionally, the experimental reproducibility can be affected by the lot-to-lot variability of Matrigel that is produced from mouse sarcoma (Murrow et al., 2017). In this respect, well-defined synthetic matrix, such as PEG-based hydrogel, can replace Matrigel as the starting housing matrix for alveolar organoids.

Moreover, how AEC2 cells influence the niche in response to lung injury can be explored with alveolar organoids. One example is the immune response from AEC2 cells elicited by microbial infections, such as *M. tuberculosis* and coronavirus (Qian et al., 2013; Ryndak and Laal, 2019; Li et al., 2020). The current outbreak of COVID-19 highlights the importance of understanding the development of coronavirus-caused pneumonia (Malta et al., 2020; Zhou et al., 2020). A recent report shows that, upon infecting the lung explant, SARS-CoV induces the expression of IFNs in 48 h, but not SARS-CoV-2, despite that the replication of SARS-CoV-2 is more efficient than SARS-CoV (Chu et al., 2020). These results lead to a question: what could be the underlying mechanism for the differential immune responses to these two coronaviruses, which share 79% sequence identity and infect AEC2 cells (Chu et al., 2020; Zhou et al., 2020)? In addition, it remains unclear how coronaviruses exit the cell (Fehr and Perlman, 2015). The secretory system for surfactant proteins in AEC2 cells could be utilized by SARS-CoV-2; Alternatively, the virus may have a unique pathway for exit, leading to reduced production of surfactant proteins and destruction of alveolar homeostasis. Alveolar organoids would be useful models to address these questions and to study the development of COVID-19 *in vitro*, complementary to animal models. Several reports have shown that airway organoids and intestinal organoids are successfully infected with influenza virus and MERS-CoV, respectively, by incubating the virus with organoids or microinjection of the virus into the inner lumen (Zhou et al., 2017, 2018; Hui et al., 2018; Bui et al., 2019; Sachs et al., 2019). We anticipate that similar methodology can be applied with alveolar organoids to investigate how AEC2 cells respond to coronavirus infection and reshape the niche.

## Concluding Remarks

Although not every aspect of alveoli within the lung can be fully recapitulated in organotypic culture, alveolar organoids help to dissect the role of the niche in AEC2 self-renewal and differentiation, thereby bridging the gap between *in vivo* model and *in vitro* culture. We envision that application of these model systems in combination will bring more insight to the development of lung diseases.

## Author Contributions

DL and HL conceived, designed, and wrote the manuscript.

## Conflict of Interest

The authors declare that the research was conducted in the absence of any commercial or financial relationships that could be construed as a potential conflict of interest.
