# Corrigendum: Dissecting the Niche for Alveolar Type II Cells With Alveolar Organoids

**DOI:** 10.3389/fcell.2021.684641

**Published:** 2021-05-20

**Authors:** Danying Liao, Huaibiao Li

**Affiliations:** ^1^Department of Haematology, Union Hospital, Tongji Medical College, Huazhong University of Science and Technology, Wuhan, China; ^2^Institute of Reproductive Health, Tongji Medical College, Huazhong University of Science and Technology, Wuhan, China

**Keywords:** lung, alveolar epithelium, alveolar type II epithelial cell, alveolar stem cell, alveolar organoid, Wnt pathway, FGF pathway, coronavirus

In the original article, there was a mistake in Supplementary Table 1 as published. In the original article, the correspondence author inappropriately included a table of an unpublished manuscript. The corrected Supplementary Table 1 appears below.

**Supplementary Table 1:** Organotypic culture methods of AEC2 cells.

**Supporting cells**	**Culture method**	**GFs/inhibitors included in basic medium**	**References**
Primary lung Fibroblasts	AEC2 cells were cultured on top of a single layer of fibroblasts in Matrigel matrix	–	Sucre et al., 2018
Primary fibroblasts or fibroblast cell line	AEC2 cells were mixed with supporting cells in Matrigel/culture medium, seeded into transwell insert for coculture. Culture medium supplemented with growth factors and inhibitors was changed regularly for organoid growth.	Y-27632, ITS, SB431542	Chen et al., 2012; Barkauskas et al., 2013; Zacharias et al., 2018
Lung mesenchymal cells		ITS	McQualter et al., 2010
lung endothelial cells		ITS	Lee et al., 2014
CD45+;F4/80+ Macrophages		ITS, EGF, KGF, FGF2, HGF	Lechner et al., 2017
Supporting cell-free	AEC2 cells were embedded in Matrigel matrix for culture. Growth factors and inhibitors were added into the culture medium to stimulate the growth of AEC2 cells.	Jagged-1,Noggin, SB431542,CHIR-99021, KGF, Y-27632	Shiraishi et al., 2019a,b
		A83-1, Noggin, Rspo1, Wnt3a, EGF, KGF, FGF10, Y-27632	Weiner et al., 2019

*ITS, insulin, transferrin and selenium; KGF, keratinocyte growth factor; EGF, epidermal growth factor; FGF2, fibroblast growth factor 2; FGF10, fibroblast growth factor 10; HGF, Hepatocyte growth factor; Rspo1, R-spondin 1*.

The authors apologize for this error and state that this does not change the scientific conclusions of the article in any way. The original article has been updated.

