# Supplementary material for: Dissecting the Niche for Alveolar Type II Cells With Alveolar Organoids
Source: Front Cell Dev Biol. 2020 Jun 4;8:419. doi: 10.3389/fcell.2020.00419 (PMC7287157; doi:10.3389/fcell.2020.00419)
Supplement: Supplementary file 1 [file Table_1.docx]

**Supplementary Table 1** Organotypic culture methods of AEC2 cells

| **Supporting**  **cells** | **Culture method** | **GFs/inhibitors included in basic medium** | **References** |
| --- | --- | --- | --- |
| Primary lung fibroblasts | AEC2 cells were cultured on top of a single layer of fibroblasts in Matrigel matrix | - | Sucre et al., 2018 |
| Primary fibroblasts or fibroblast cell line | AEC2 cells were mixed with supporting cells in Matrigel/culture medium, seeded into transwell insert for coculture. Culture medium supplemented with growth factors and inhibitors was changed regularly for organoid growth. | Y-27632, ITS, SB431542 | Chen et al., 2012; Barkauskas et al., 2013; Zacharias et al., 2018 |
| Lung mesenchymal cells |  | ITS | McQualter et al., 2010 |
| lung endothelial cells |  | ITS | Lee et al., 2014 |
| CD45+;F4/80+ Macrophages |  | ITS, EGF, KGF, FGF2, HGF | Lechner et al., 2017 |
| Supporting cell-free | AEC2 cells were embedded in Matrigel matrix for culture. Growth factors and inhibitors were added into the culture medium to stimulate the growth of AEC2 cells. | Jagged-1,Noggin, SB431542,CHIR-99021, KGF, Y-27632 | Shiraishi et al., 2019a,b |
|  |  | A83-1, Noggin, Rspo1, Wnt3a, EGF, KGF, FGF10, Y-27632 | Weiner et al. 2019 |

ITS, insulin, transferrin and selenium; KGF, keratinocyte growth factor; EGF, epidermal growth factor; FGF2, fibroblast growth factor 2; FGF10, fibroblast growth factor 10; HGF, Hepatocyte growth factor; Rspo1, R-spondin 1
